# “When everyone is responsible, no one takes responsibility”: exploring pediatric physiotherapy services in Israel

**DOI:** 10.1186/s13584-024-00597-w

**Published:** 2024-02-27

**Authors:** Nilly Waiserberg, Tuvia Horev, Paula Feder-Bubis

**Affiliations:** 1 Department of Health Policy and Management, Faculty of Health Sciences, Ben-Gurion University of the Negev, David Ben Gurion Blvd 1, P.O.B. 653, Beer-Sheva, Israel; 2 Department of Physical Therapy, School of Health Professions, Faculty of Medicine, Tel Aviv University, Chaim Levanon Street, P.O.B. 39040, Tel Aviv, Israel; 3 Department of Health Policy and Management, Guilford Glazer Faculty of Business and Management, Ben-Gurion University of the Negev, David Ben Gurion Blvd 1, P.O.B. 653, Beer-Sheva, Israel; 4 Department of Health Policy and Management, Faculty of Health Sciences and Guilford Glazer Faculty of Business and Management, Ben-Gurion University of the Negev, David Ben Gurion Blvd 1, P.O.B. 653, Beer-Sheva, Israel

**Keywords:** Children with motor disabilities, Physiotherapy services, Policymakers’ perspectives, Qualitative research

## Abstract

**Background:**

According to Israel's National Health Insurance Law (1994), the Ministry of Health is responsible for the provision of health services in the country including physiotherapy services; moreover, the Special Education Law (1988), stipulates that physiotherapy services for children with motor disabilities, as well as other allied health services, are provided by the Ministry of Education in educational settings. Thus, children with motor disabilities are entitled PT services under two different laws by two different ministries.

**Method:**

To describe the physiotherapy services for children with motor disabilities and examine how policymakers view these services, we conducted a qualitative study including in-depth semi-structured interviews with 10 policymakers from the Ministry of Health and the Ministry of Education, and the national directors of physiotherapy services from three of the four health maintenance organizations in Israel.

**Results:**

Study results indicate that there is an array of physiotherapy services and providers. Despite the regulation of these services for children with motor disabilities, uncertainty and lack of knowledge were found about various issues. Therefore, the thematic analysis was structured around four descriptive questions: Where do the children receive physiotherapy? Who is eligible for physiotherapy treatment and who receives treatment? What interventions do children with motor disabilities receive? Who provides therapy for children with motor disabilities?

**Conclusions:**

Policymakers are dubious regarding the provision of these services, questioning whether children with motor disabilities receive physiotherapy services according to their needs. In addition, the abundance of suppliers does not necessarily improve the quality of services provided to children with motor disabilities, which may ultimately harm their developmental potential.

## Introduction

Children with lifelong motor disabilities such as cerebral palsy, muscular dystrophy and neurogenetic disorders usually receive tailored health services from birth to adulthood according to their needs. Their complex developmental and medical condition usually requires the involvement of several health professionals whose collaboration is unique given these needs. Physiotherapy is the most commonly used intervention for these children [1]. They require an appropriate therapy to help promote their functioning, participation, prevent secondary impairments and increase their capabilities and potential to live independently. The populations, organizational settings, and characteristics and contexts of the service delivery of this therapy vary a great deal [2]. The main settings in which physiotherapists treat children with disabilities are in schools, homes, hospitals, and outpatient and/or community settings [3, 4].

In the last few decades, there has been a change in physiotherapy theory and practice [5], particularly pediatric physiotherapy. The International Classification of Functioning, Disability and Health framework (ICF) [6] represents a paradigm shift from a medical to a bio-psychosocial approach that has been widely adopted by rehabilitation research and in clinical settings worldwide, including in Israel [7, 8]. Similarly, pediatric physiotherapy services have shifted to holistic family-centered models [9, 10], and many medical center-based physiotherapy services for children have been replaced by physiotherapy services in more natural environments such as the home or school [11, 12]. Support for this approach comes from the publication of the ICF-Children and Youth from the World Health Organization [13], which helps identify the strengths and needs of the child and the family, along with environmental considerations. Thus, in many countries, along with medical settings, treatment in educational settings plays a significant role in the provision of therapy to children with disabilities [14].

In Israel, children with motor disabilities are entitled to physiotherapy services under two different laws administered by two different government ministries. According to the National Health Insurance Law (NHIL), one of the four health maintenance organizations (HMOs) must provide a basic set of health services including physiotherapy, monitored by the Ministry of Health. All citizens are insured by a mandatory health insurance and can meet with a physiotherapist as part of their rehabilitation services, in acute or chronic care settings or as part of child development services [15]. Every child is entitled to child development services. A child with a disability due to a disease that impairs the central and peripheral nervous system or the musculoskeletal system and causes persistent functional disability is defined as *somatic* and entitled to physiotherapy services as needed and without limitation up to age 18. A child who is not defined as having a *somatic* disorder is entitled to receive an unlimited number of therapy sessions up to 3 years of age, decreasing till 9 sessions per year up to age 9 [15].

The second law dealing with physiotherapy, the Israeli Special Education Law (1988), specifies that physiotherapy services for children with motor disabilities be provided by the Ministry of Education within educational settings. These services are part of the additional services to children ages 3–21 with special needs, along with occupational therapy, speech therapy and other services according to each child’s needs. The frequency, intensity and type of intervention are determined collaboratively by the school team as part of the personal program of the child, and according to the ability of the system to provide them [16]. It should be noted that children who receive physiotherapy as part of special education services do not lose their eligibility to receive physiotherapy from the healthcare system. Our aim was to describe physiotherapy services for children in Israel as perceived by policymakers and to learn about their perception of these services implementation.

## Methods

### Study design

We conducted an exploratory qualitative study including in-depth semi-structured interviews with 10 policymakers in the Ministries of Health and of Education, as well as national and regional directors of physiotherapy services from three of the four HMOs in Israel.

### Recruitment of participants

Israel is a small country, particularly within professional circles. Therefore, in recruiting our critical case participants [17], we assured them of their anonymity. To do so, we will provide limited descriptive information about them. Almost all were women (9 of 10), and most were physiotherapists (8 of 10). All participants served full-time in their policymaker position and engaged with the field daily. We approached these interviewees by e-mail explaining the purpose of the study, followed by a phone call to schedule the interview at a place and time convenient for them. Everyone we approached agreed to participate. Most interviews took place in the office of the interviewees during working hours. All participants signed a written informed consent.

### Data collection and data analysis

In-depth interviews were conducted to obtain complete, detailed accounts of the participants’ experience, providing rich data, and allowing the participants to share their perceptions in detail [18]. The first author conducted all of the interviews to ensure the consistency and reliability of the data collection. A semi-structured interview guide was used for each interview (see Table 1). The first author is a physiotherapist. This research was part of her PhD thesis supervised by the second and third authors, a health policy specialist and medical sociologist, respectively.

**Table 1:** Interview protocol

	Question	Probe
1	Tell me a little about yourself and your work	Training, role Place/jobs today and in the past Contract with pediatric physiotherapy
2	What are the settings in which children with motor disabilities receive physiotherapy intervention in Israel?	Who is the regulator in each setting? Are you satisfied with the services children receive? If not, why not? What are the advantages, challenges and facilitating factors?
3	What do you think is an ideal physiotherapy treatment for a child with a motor disability?	What is the focus of physiotherapy intervention? What are the goals? Which children need physiotherapy intervention? What/where do you think is the best setting for physiotherapy? In an educational setting? At home? In a medical setting? How can we determine “best practices” for children?
4	Can you compare physiotherapy practices in medical settings with school-based physiotherapy?	Are the treatment goals the same? Similarities and differences in clinical reasoning
5	How does pediatric physiotherapy professional development happen?	What are the opportunities for knowledge transfer? Availability and accessibility of professional courses? Seminars? What sort of knowledge is important to a pediatric physiotherapist? What are the professional development opportunities in the education system/healthcare organizations?
6	What is a professional pediatric physiotherapist?	How is professionalism built? How is professionalism expressed?
7	Do you think children with motor disabilities receive the physiotherapy services they are entitled by law and according to their needs?	

All interviews were conducted in Hebrew and lasted 46–103 min. The atmosphere was positive and relaxed, and the interviewees took the time requested for the interview and spoke honestly.

Interviews were transcribed verbatim and thematic analysis was performed [19]. We ensured the trustworthiness of the study by using the constant comparison method, peer debriefing, reflexivity, and audit [20]. We analyzed the transcripts as part of an iterative process involving several steps. We began by familiarizing ourselves with the material by reading the transcripts, noting salient issues and potential patterns within the data. We followed this approach after each interview. Once familiar with the material, we began coding it and identifying the themes. The analysis continued with the definition, revision, and naming of the themes [21]. Data saturation was reached after finalizing the analysis of these interviews.

The institutional review board of [anonymized] University’s Human Subjects Research Ethics Committee approved this study: number 1701-1.

## Results

**Fig. 1:**
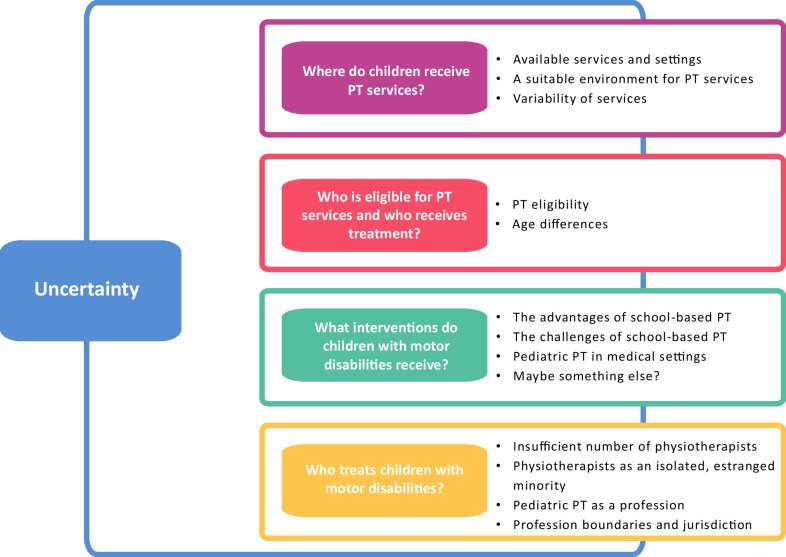
Themes and subthemes

The interviews with policymakers revealed the overarching theme of *uncertainty*. Despite the existing laws that establish the entitlement of children with motor disabilities to physiotherapy services, the participants expressed uncertainty as to whether these services are supplied. This uncertainty led to organizing the themes as four descriptive questions: *Where do children receive physiotherapy? Who is eligible and who receives physiotherapy services? What interventions do children with motor disabilities receive?* and *Who treats children with motor disabilities?* Fig. 1 describes the four themes and subthemes, including the overarching theme.

The array of pediatric services offered to children with motor disabilities was described as broad and generous, but the interviewees had doubts about their implementation. They were uncertain as to whether these children indeed receive the physiotherapy they need. Participants stated that many agencies provide pediatric physiotherapy. In the public sector, such services are available through the healthcare and education systems. Services are also available through the private sector and through non-governmental organizations (NGOs). Although the educational environment was described as an opportunity to provide service in the children's natural environment, it was not clear whether they do receive physiotherapy there, and if so, of what quality. The interviewees described the healthcare system as providing selected services, aimed primarily at younger children. The interviews revealed that despite the laws aimed at standardization, their interpretation and implementation vary, leading to gaps in the accessibility and availability of therapy. Responsibility for providing services is divided among multiple suppliers, which ultimately results in dependence on a single person—the service provider or receiver. As one interviewee noted anxiously: "Actually, it is everyone's responsibility, so when everyone is responsible, no one takes responsibility" (#7).

The policymakers questioned not only the provision of the services but also the service providers, namely, pediatric physiotherapists. They questioned their professionalism and professionalization. Their descriptions revealed that there are not enough pediatric physiotherapists. In addition, they are a minority group both among all physiotherapists and within the various systems. Professional boundaries were also mentioned as a challenge, including ambiguity surrounding core professional areas and the various factors that erode these boundaries, resulting in some of the core roles that define pediatric physiotherapy being taken over by other professions.

### Where do children receive physiotherapy services?

#### Available services and settings

**Fig. 2:**
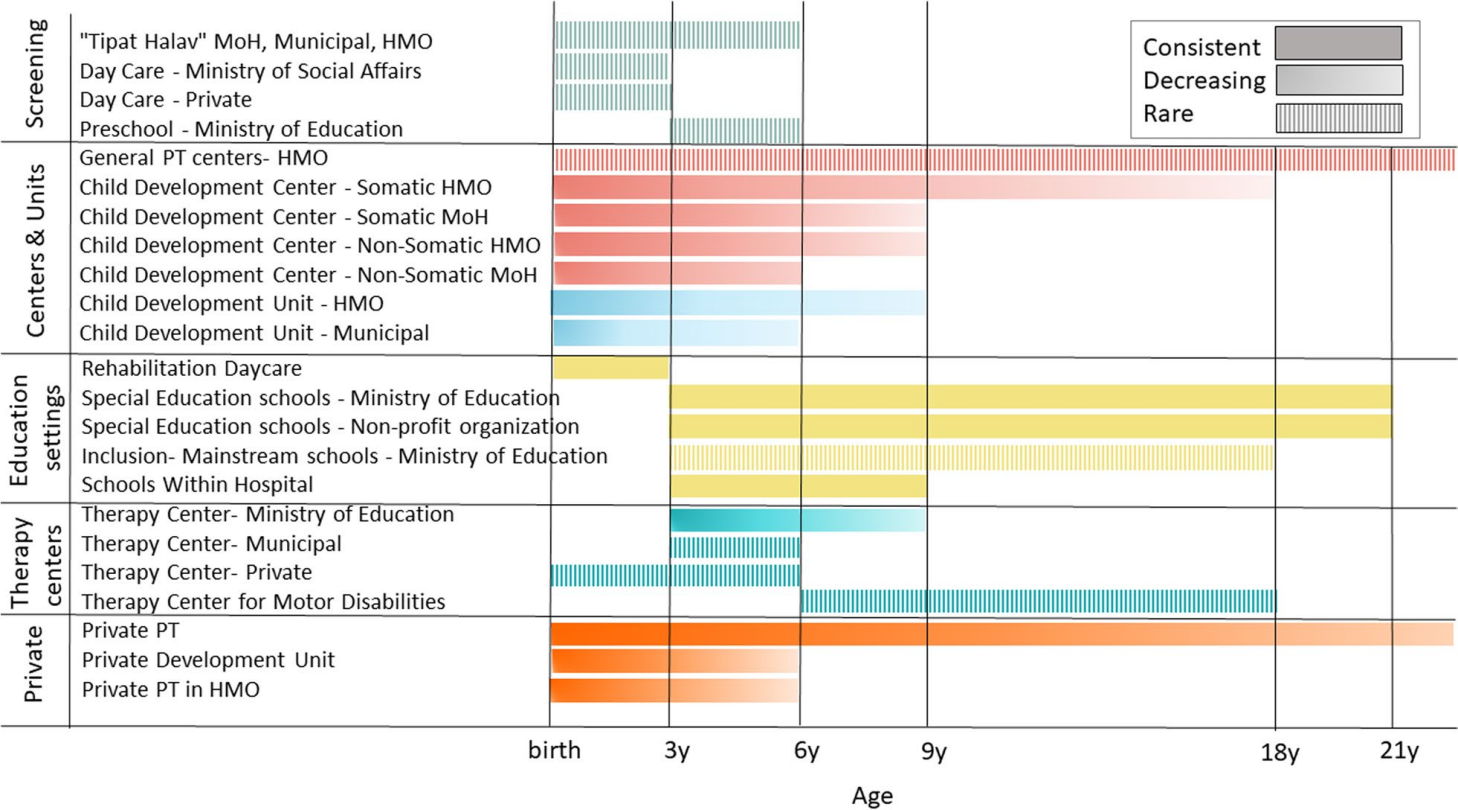
The range of physiotherapy services reported by policymakers, according to age and setting. *Notes*: The vertical axis indicates the type of treatment whereas the horizontal axis indicates the availability of interventions by age. Decreased opportunities were reported as children get older. MoH- Ministry of Health, HMO- health maintenance organizations

As the participants reported, there are many settings that provide pediatric physiotherapy services in Israel. Different services were reported by different participants. Figure 2 shows the range of services reported according to age and setting, including screening services, medical institutions, educational settings and private clinics. Many centers and institutes are part of the public sector: the Ministries of Health, Education, and Social Affairs, municipal institutes and the HMOs. Some belong to or are managed by nonprofit organizations such as Jewish, Christian or Muslim religious organizations, and organizations for children with autism and cerebral palsy. There are also private pediatric physiotherapy clinics. According to the policymakers, the availability of physiotherapy services varies by region of the country, and between and within HMOs, municipalities and the school system.

Only in a few centers or public programs are physiotherapists involved in the screening for developmental delays, posture and movement limitation, and only up to age 6. There are various child development centers and units across the country operated by the HMOs, the Ministry of Health, hospitals, and municipal and private organizations. Although required by NHIL, they mainly serve infants and young children. As one participant said:

Older children should also be treated in child development institutes. They are supposed to … Little kids and babies definitely do, but I know the older ones receive less, I guess (# 3).

Children sometimes receive physiotherapy in general orthopedic physiotherapy centers. One participant commented:

There is a physiotherapist at the general physiotherapy institute whom I have known for 30 years. He typically works with adult orthopedic patients, but he also sees children there, despite never taking any courses specifically related to pediatric physiotherapy and never having worked at a child development institute (#8).

According to all policymakers, most treatment for children with motor disabilities is provided in special educational settings. However, as one participant claimed, physiotherapy services for children in inclusion programs in mainstream schools are inconsistent and vary greatly:

Children with motor disabilities receive physiotherapy in special education schools. I don't know whether to say that it is full, but certainly in special education schools there are physiotherapists who take care of these children... those in mainstream education should also receive physiotherapy at school, but it doesn't always happen. And if not in school, then where do they get it, I don't know (#5).

Participants also reported a range of private physiotherapy services for children provided at home, in private clinics, centers, and various combinations of all of the above such as private physiotherapists working in public clinics.

#### A suitable environment for physiotherapy services

Doubts were expressed about the suitability of where treatment is provided, in both schools and healthcare settings. "I would like children to receive physiotherapy treatment in appropriate settings for therapy. Unfortunately, it is not always like that" (#5). Most participants described the lack of suitable physical environments for therapy and lack of required equipment in the education system: "The question is, do they [in education settings] have a suitable environment to provide physiotherapy intervention?" (#3). In healthcare settings, participants questioned the appropriateness of a treatment setting for all ages: "It is very unpleasant for a 13-year-old to come to the child development center. Not because we are not respectful, but the atmosphere is geared towards babies and very young children" (#4).

#### Variability of services

All participants reported great variation across regions in both the healthcare organizations: "I don’t know where therapy takes place in each of the HMOs; I don’t know because it is different for each one and different in every district" (#6) and the educational institutions: "There are 67 regional institutes around the country, 67 different models of providing services for children with motor disabilities, it's like 67 different countries" (#9). Some participants stated that the root of this inconsistency lay in the difficulties in access to services in certain areas, and policy differences within school districts and HMOs.

### Who is eligible for physiotherapy services and who receives treatment?

Despite consensus regarding eligibility for physiotherapy treatment, all participants reported that there is no clear policy or certainty regarding the supply and funding of this treatment. They indicated that children do not receive physiotherapy treatment according to their needs, as stipulated by the law.

#### Physiotherapy eligibility

Participants shared their impression that children do not receive the treatment to which they are entitled or require. Concern was expressed regarding children with minor motor limitations such as Developmental Coordination Disorder (DCD) or Autistic Spectrum Disorder who are not entitled to physiotherapy services like other children with motor disabilities: "Children with DCD are not eligible for physiotherapy at school or child development centers after the age of 6" (#1).

Despite the laws and generous funding, when asked whether children receive the physiotherapy services they need or the treatment they deserve under the laws, the answer was:

No, 100% no! (#10).

No, I don’t think so, let's say… I don’t think that there are enough institutes… (#3).

I can’t say… not fully… we don’t have enough physiotherapists and other issues that make it complicated… (#5).

#### Age differences

As Fig. 2 indicates, there are fewer physiotherapy services as children grow older. Those that are provided generally occur in special education settings or the private sector. Participants were concerned about older children who do not receive physiotherapy: "I hope older children receive services at school. I hope, but I’m not sure it happens, and I am not sure it’s enough" (#1). They were also concerned that the developmental centers, although responsible for providing these services, serve only young children.

I do not know of many child development centers that provide physiotherapy services for children over 6 or 9 years of age. I assume that older children with motor disabilities receive the physiotherapy services elsewhere… I really don’t know where (#7).

The participants’ concerns were not limited to children. They were also worried about the availability of physiotherapy services for adults with motor disabilities, who age-out of the special education system after 21. “A person isn’t cured of cerebral palsy, it is for life, but ‘the system’ does not assist those over 18… or 21 years. Their independence and ability to function independently often depend on physiotherapy…” (#3).

### What interventions do children with motor disabilities receive?

The participants were also uncertain about the content of the physiotherapy interventions for children with motor disabilities in various settings.

#### The advantages of school-based physiotherapy

School-based physiotherapy is provided in a child's natural environment, in accordance with the principles of the ICF. The aim of the treatment is to facilitate the child's participation in school, rather than in separate clinical settings. Although all interviewees supported the idea of physiotherapy intervention within educational settings, most of them questioned whether this approach is actually being implemented:

I think there is great value in having physiotherapy services in a natural environment, such as the school. Because children spend six, eight hours in this environment, treatment has to be adapted to where they live. But I have no idea of what is really going on there (#10).

Another advantage of providing physiotherapy in educational settings is that the physiotherapists can accompany and monitor the child over time. By doing so, they can detect any changes that may require attention, particularly during adolescence when some level of decline is expected.

During puberty, there is often a decrease in physical function, which can lead to a reduced quality of life. It's important to catch these issues early and prevent them, and this is where the educational framework comes in. By working in a school, physiotherapists have the opportunity to monitor the children's progress over time and detect any signs of decline before they become more serious (#1).

#### The challenges of school-based physiotherapy

Despite the advantages of providing physiotherapy services in schools, the participants also noted various difficulties and challenges associated with working as healthcare professionals (especially physiotherapists) within the education system: "It may be that the education system does not exactly understand what the role of a physiotherapist is" (#6). Specifically, they indicated that the education system makes providing the expected standard of practice difficult due to a lack of access to professional resources and artifacts, as well as a lack of specific continuing education among the physiotherapists who work there. "I'm not sure how physiotherapy treatment is provided in those [education] settings, or what kind of post-graduate training physiotherapists receive there. I'm not sure if there are any regulations to ensure that physiotherapists are practicing effectively there" (#4).

#### Pediatric physiotherapy in medical settings

Policymakers also raised doubts about the suitability of the provision of physiotherapy services to children with motor disabilities in healthcare settings. Most of them noted that physiotherapists in child development centers might be more familiar with working with children who have mild developmental delays and less experienced with children who have motor disabilities or more complex, long-standing problems: "Physiotherapists working in child development centers do not commonly see children with motor disabilities; they don’t always know what assistive devices to provide them; it is not their expertise " (#8). The nature of the treatment in healthcare settings was also unclear. As stated, in some HMOs, physiotherapy for children is provided in general physiotherapy centers, where none of the therapists are trained to treat children with motor disabilities.

#### Maybe something else?

A few participants suggested that other, currently unavailable treatment settings might be more appropriate for children with motor disabilities. They highlighted the need for leisure facilities that could offer a more suitable response to the needs of individuals with disabilities and expressed the need for physiotherapists to be involved in such settings.

It's possible that there are other ways to meet the motor needs of children with disabilities… One possibility could be to provide adapted physical activity in community settings, such as having a physiotherapist available for intervention during leisure activities (#3).

### Who treats children with motor disabilities?

All policymakers expressed serious concerns about several issues including the insufficient number of physiotherapists, their status as a minority in the education system, their clinical isolation and position as strangers in schools, their lack of supervision, and doubts about pediatric physiotherapy as a profession, creating a vacuum filled by others.

#### Insufficient number of physiotherapists

All participants pointed out that there is a shortage of pediatric physiotherapists in all sectors, particularly in the education system: "There are not enough pediatric physical therapists, there are not enough physiotherapists in the educational system and in the HMOs" (#3). They gave several reasons for their small number including their reluctance to work for low wages and limited benefits, and “lack of awareness of the need for physiotherapy in school systems" (#5).

#### Physiotherapists as an isolated, estranged minority

Policymakers described pediatric physiotherapists as a minority, a major issue in the education system: "You are an outsider there… and you are alone… most of the other staff are teachers, so it’s complicated…" (#7). The participants described the pediatric physiotherapists as a minority within the profession, the physiotherapy union, and the various systems. As such, these physiotherapists feel isolated. Pediatric physiotherapists also have insufficient training and fewer professional development opportunities compared to other areas of physiotherapy practice: "There are only a few post-graduate courses in pediatrics, while in orthopedics and neurology, they are countless" (#10). Finally, the participants also noted that the physiotherapists feel like strangers in the education system:

Physiotherapy is secondary in the education system, even within the health professions… everyone needs to talk, so they [the students] receive speech therapy… everyone needs to write and hold a pencil, so who will teach them? An occupational therapist… and due to various misconceptions, the OT will also examine motor performance. So why bring in another clinician? (#9).

#### Pediatric physiotherapy as a profession

Questions, doubts and uncertainties were expressed regarding pediatric physiotherapy as an area of practice and its professional development. Most, including participants who are not physiotherapists by training, described the profession as "decaying," noting a lack of advocacy for the profession. “You should love yourselves. If a physiotherapist tells me ‘I refer that child to an occupational therapist’ it means you lost your trust in yourselves, start loving yourselves” (#6). Negative attitudes were also expressed about the public system, academia, and the physiotherapy union, with one interviewee saying: “My professional society does nothing for me" (#7). Interviewees stated that in the absence of regulation and guidance, pediatric physiotherapists must seek their own professional development: "Someone needs to define what constitutes professional development for pediatric physiotherapists… that should be determined by the physiotherapy Association, or by the government; it currently does not exist" (#6).

#### Professional boundaries and jurisdiction

Participants expressed concerns about the internal and external erosion of professional boundaries.

There is a big problem with physiotherapy professional boundaries: where do they begin and end, who needs physiotherapy, who should be referred to a physiotherapy… I mean, it is unclear to the public but also to therapists, when should a physiotherapy or OT be consulted?... I think this ambiguity, along with a halo surrounding occupational therapy, challenges the profession (#6).

The participants also noted that other professionals such as occupational therapists, non-professionals such as developmental coaches, and others such as art therapists and sports coaches provide treatments that include core physiotherapy skills in their practice with children with motor disabilities. In addition to others who adopt similar skills as the core skills of physiotherapists, a concern was also expressed about the erosion of the profession due to the lack of consistency between therapists and “unprofessional” physiotherapists:

At least inside our profession there should be some consensus, some uniformity. We have enough fighting against all other occupations... So at least we, the physiotherapists, should be professional; we must provide families with realistic and attainable goals (#2).

## Discussion

This study described pediatric physiotherapy services in Israel from the perspectives of policymakers from the various organizations responsible for providing these services. Children are entitled to these services according to the NHIL and the Special Education Law; the latter is similar to the Individuals with Disabilities Education Improvement Act [22, 23].

Despite the clear regulations regarding the provision of physiotherapy services for children with motor disabilities, policymakers were dubious regarding their implementation, questioning whether these children receive physiotherapy services according to their needs. Their lack of knowledge about this implementation indicates pervasive uncertainty among policymakers that can have a ripple effect on physiotherapists and the parents of children with disabilities. Thus, having laws that require the provision of physiotherapy services does not ensure that children will receive the services they need.

These findings are in line with those of Jacob and Parag's qualitative study regarding differences in the provision of physiotherapy services in Israel. Namely, physiotherapy services unequal availability, lack of availability in periphery areas of the country, and the wide variability in the competency of the physiotherapists [24]. Similarly, based on data from 47 countries, including Israel, Camden et al. noted lack of direct access to physiotherapy services, insufficient specialized physiotherapy, and financial and geographical issues as the leading barriers to access to pediatric physiotherapy services [25].

While there are numerous providers of physiotherapy services from public, private and not-for-profit organizations, their abundance does not necessarily translate into appropriate services for children. Furthermore, when there are multiple providers, it is difficult to navigate the systems and track the nature of practice and service. The absence of an integrated system and clear policies were cause for concern for policymakers and physiotherapy directors. Coordinated care is essential to improve clinical outcomes and navigate the healthcare and education services within and across settings to help children receive care where and when needed [26, 27].

The participants also reported that there are fewer opportunities to see a physiotherapist as children grow older. This finding is of particular concern because there is a natural decline in motor capacity and performance in older children and adolescents with motor disabilities [28]. They suffer from pain, a decreasing range of motion, and engage in little physical activity [29]. Inactivity has long-term negative health consequences [30] and results in early loss of function [31, 32]. People living with disabilities (PLWD) have poorer health than the general population and are at a greater risk of injury and of developing non-communicable chronic diseases and age-related health conditions at earlier ages [33]. There is increasing awareness among policymakers of the need of PLWD to participate in physical activity through effective interventions [34]. Physiotherapists can provide long-term interventions to PLWD throughout the stages of their lives, resulting in a positive impact [35]. Given that the frequency and access to physiotherapy services decrease dramatically as children grow older, it is necessary to create appropriate frameworks and ensure access to these services that will continue into adulthood [36, 37]. Policymakers should develop physical activity action plans involving physiotherapy services to promote health among children and adults living with physical disabilities [34].

According to policymakers, physiotherapy is provided most consistently in special educational settings. Fundamental questions were raised regarding professional practice in atypical settings [38, 39]. Previous literature has indicated that physiotherapists commonly treat children with disabilities in schools [3, 4], even though they struggle to have a voice or identity in school systems [40]. Participants also expressed concerns about school-based physiotherapists’ professionalism, clinical isolation in an unfamiliar work setting and lack of supervision. Limited access to professional resources and artifacts and lack of representation of expertise can blur occupational boundaries and impact professional outcomes [41, 42].

The lack of a clear and uniform policy for implementing the laws, coupled with external factors that erode the boundaries of the profession, poses a significant threat to the provision of adequate care for children with motor disabilities. The joint responsibility of two separate systems for the provision of pediatric physiotherapy services challenges the overall provision of these services due to the lack of formal coordination between these two systems. Additionally, with a large number and wide variety of providers, it can be challenging for patients and their families to navigate between the systems and to maintain continuity of care [26, 27].

The uncertainty and variability of pediatric physiotherapy services depicted in this study are based on policymakers' perspectives, which might be a limitation because managers in the field, physiotherapists, and the families might perceive them differently. Their perspectives should be addressed in further studies. Since this is an exploratory research, further studies need to focus on the actual provision of services and their coordination, the scope of practice of pediatric physiotherapy in different settings, and on the development of pediatric physiotherapists' professionalism. These studies might improve the implementation of the existing laws.

## Conclusions

Given the array of services and providers of pediatric physiotherapy, better coordination of care, services and suppliers is necessary to improve physiotherapy services for children with motor disabilities in Israel. Policymakers in the systems that provide these services should engage also in policymaking about coordination and cooperation between systems.

On the professional level, external factors that erode the boundaries of physiotherapy professional practice threaten the continued existence of physiotherapy as a discipline with defined standards of clinical practice. Jointly with professional organizations, policymakers need to strengthen and further develop pediatric physiotherapists' professional practice. This professionalism will contribute to better health outcomes among children with motor disabilities.

## Data Availability

The data presented in this study are available on request from the corresponding author. The data are not publicly available due to privacy restrictions.
